# Chronic kidney disease is related to impaired left ventricular strain as assessed by cardiac magnetic resonance imaging in patients with ischemic cardiomyopathy

**DOI:** 10.1007/s00392-023-02346-6

**Published:** 2023-12-11

**Authors:** Rosalia Dettori, Andrea Milzi, Richard Karl Lubberich, Kathrin Burgmaier, Sebastian Reith, Nikolaus Marx, Michael Frick, Mathias Burgmaier

**Affiliations:** 1 Department of Internal Medicine I, University Hospital of the RWTH Aachen, Pauwelsstraße 30, 52074 Aachen, Germany; 2 Faculty of Applied Healthcare Science, Deggendorf Institute of Technology, Deggendorf, Germany; 3 Department of Pediatrics, University Hospital Cologne and Faculty of Medicine, University of Cologne, Cologne, Germany; 4 Department of Internal Medicine III, St. Franziskus Hospital, Münster, Germany

**Keywords:** Cardiac magnetic resonance imaging, Chronic kidney disease, Ischemic cardiomyopathy, LV strain, Myocardial scar

## Abstract

**Introduction:**

Chronic kidney disease (CKD) is an important cardiovascular risk factor. However, the relationship between CKD and myocardial strain as a parameter of myocardial function is still incompletely understood, particularly in patients with ischemic cardiomyopathy (ICM). Cardiac magnetic resonance imaging (CMR) feature tracking allows to analyze myocardial strain with high reproducibility. Therefore, the aim of the present study was to assess the relationship between CKD and myocardial strain as described by CMR in patients with ICM.

**Methods:**

We retrospectively performed CMR-based myocardial strain analysis in 89 patients with ICM and different stages of CKD, classified according to the KDIGO stages. In all patients, global longitudinal strain (GLS), global circumferential strain (GCS) and global radial strain (GRS) analysis of left ventricular myocardium were performed. Furthermore, segmental longitudinal (SLS), circumferential (SCS) and radial strain (SRS) according to the AHA 16/17-segment model was determined.

**Results:**

Creatinine levels (GLS: *r* = 0.46, *p* < 0.001; GCS: *r* = 0.34, *p* = 0.001; GRS: *r* = − 0.4, *p* < 0.001), urea levels (GLS: *r* = 0.34, *p* = 0.001; GCS: *r* = 0.30, *p* = 0.005; GRS: *r* = − 0.31, *p* = 0.003) as well as estimated glomerular filtration rate (GLS: *r* = -0.40, *p* < 0.001; GCS: *r* = − 0.27, *p* = 0.012; GRS *r* = 0.34, *p* < 0.001) were significantly associated with global strains as determined by CMR. To further investigate the relationship between CKD and myocardial dysfunction, segmental strain analysis was performed: SLS was progressively impaired with increasing severity of CKD (KDIGO-1: − 11.93 ± 0.34; KDIGO-5: − 7.99 ± 0.38; *p* < 0.001 for KDIGO-5 vs. KDIGO-1; similar data for SCS and SRS). Interestingly, myocardial strain was impaired with CKD in both segments with and without scarring. Furthermore, in a multivariable analysis, eGFR was independently associated with GLS following adjustment for LV-EF, scar burden, diabetes, hypertension, age, gender, LV mass or LV mass index.

**Conclusion:**

CKD is related to impaired LV strain as assessed by CMR in patients with ICM. In our cohort, this relationship is independent of LV-EF, the extent of myocardial scarring, diabetes, hypertension, age, gender, LV mass or LV mass index.

## Introduction

Chronic kidney disease (CKD) is associated with an increased risk for cardiovascular morbidity and mortality [1, 2]*.* It is well known that coronary artery disease (CAD) represents one of the predominant manifestations of cardiovascular disease (CVD) in patients with CKD and its prevalence increases with deteriorating kidney function [3–5]. The high prevalence of traditional cardiovascular risk factors in patients with CKD, such as hypertension, diabetes, dyslipidemia and smoking, is not only of major importance for the development of atherosclerotic vascular disease [6, 7], but also for the progression of CKD itself [7–9]. Apart from classical risk factors, the course of CVD also depends on CKD-specific, non-classical risk factors, including proinflammatory circulatory mediators, renal hormones, enzymes and cytokines (reviewed in [7]). These complex processes may promote vascular calcification, hemodynamic alterations [10–12] and characteristic myocardial changes such as myocardial fibrosis [13] and hypertrophy, as precursors of uremic cardiomyopathy [7, 14]. However, the exact pathophysiological mechanisms of uremic cardiomyopathy are still not fully understood and further research is needed to elucidate the effects of CKD especially on myocardial structure and contractility.

Cardiac magnetic resonance (CMR) represents a well-established, non-invasive modality in the diagnosis and management of CVD*.* The high spatial and temporal resolution of CMR allows an accurate quantification of myocardial function and, in particular, of myocardial structure [15]. In the past years, myocardial strain (MS) imaging has emerged as a robust tool to analyze cardiac deformation as a marker of preclinical ventricular dysfunction prior to a decrease of ventricular ejection fraction and overt cardiovascular disease [16, 17]. In this context, myocardial feature tracking (FT) is considered a precise technique to quantify myocardial strain based on CMR cine sequences, without the need of gadolinium-based contrast agents [18–20].

Several studies analyzed the feasibility of MS in patients with altered cardiac conditions. In patients with myocardial infarction, global and segmental MS were impaired presumably as a consequence of a gradual replacement of necrotic myocardium by fibroblasts, which leads to a reduced contractility of the affected segments [18, 21]. These results are in line with the observations of Stathogiannis et al., who described alterations in regional peak systolic longitudinal and circumferential strains within areas of myocardial scar in patients with ischemic heart disease [22]. Also, other clinical conditions, such as the presence of diabetes mellitus type 2 (T2DM), have been associated with a decrease of left ventricular global strain [23]. Besides, in end-stage renal disease patients with chronic dialysis, speckle-tracking echocardiography-derived GLS has been proposed as a more reliable predictor of cardiovascular mortality than echocardiography-derived left ventricular ejection fraction (LV-EF) [24]. Previous studies already showed that increasing severity of CKD is associated with worsening MS in patients without a history of cardiovascular disease [25]. However, the association between CKD and MS in patients with ischemic cardiomyopathy (ICM) is still unclear. Thus, the aim of this study was to investigate the effects of CKD on MS as a surrogate marker of ventricular dysfunction in patients with ICM and how this relationship may be influenced by the presence of myocardial scarring.

## Materials and methods

### Patient selection

We retrospectively included a total of 89 patients with ICM (defined as heart failure in the presence of CAD and/or prior myocardial infarction) and normal kidney function or different stages of CKD, classified according to the KDIGO stages, who underwent CMR from October 2014 until October 2021 at the Department of Cardiology, University Hospital of the RWTH Aachen.

Main exclusion criterion was insufficient image quality (e.g. due to patient incompliance or irregular heart rhythm because of atrial fibrillation or premature ventricular contractions).

The registry was approved by the local ethics committee and is in accordance with the declaration of Helsinki on ethical principles for medical research involving human subjects.

### CMR data

CMR image acquisition took place on a 1.5 Tesla magnetic resonance scanner (Achieva, Philips Healthcare, Best, The Netherlands). In all cases, standard native cine balanced SSFP images were available in standard long-axis geometries as well as in short-axis orientation covering the entire ventricles. In all patients, standard late gadolinium enhancement (LGE) imaging was available. The application of intravenous gadolinium-based contrast media (gadobutrol; Gadovist ® Bayer Healthcare GmbH, Germany) in patients with kidney disease followed an internal standard operating procedure in line with the European Society of Urogenital Radiology (ESUR) guidelines on contrast media and the consensus statements from the American College of Radiology and the National Kidney Foundation [26]. Standard dosage of gadobutrol was 0.15 mmol/kg bodyweight, with a reduction to 0.1 mmol/kg bodyweight in patients with an estimated glomerular filtration rate less than 30 mL/min/1.73 m^2^.

Left ventricular ejection fraction (LV-EF) and end diastolic myocardial mass were derived from the cine short-axis stack. LV Mass index was calculated taking into account the body surface area. Extent, transmurality and location of myocardial scarring were determined according to standard operating procedures based on international post-processing recommendations [27]. For each segment, presence or absence of myocardial scar as well as transmurality were assessed. Scar burden was defined as the number of scarred segments, each weighted for its scar transmurality.

### CMR strain analysis

**Fig. 1:**
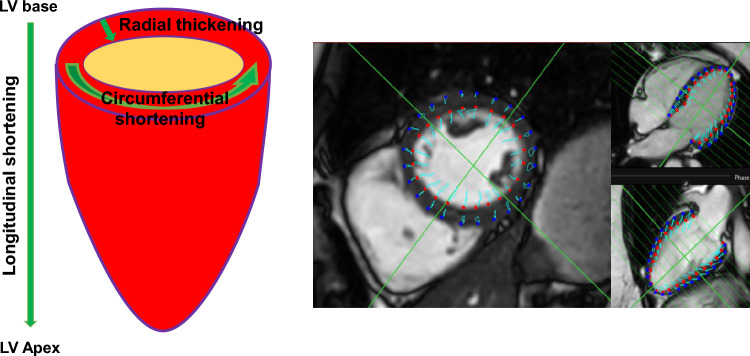
Graphic illustration of myocardial strain analysis in CMR. Left: a graphic illustration of myocardial deformation (longitudinal, circumferential, radial) of the left ventricle is shown. Right: automatic contouring of endocardium (red dots) and epicardium (blue dots) of the short-axis stack as well as of the two- and four-chamber long axes at end-diastole is shown. Subsequently, automatic propagation of myocardial borders is initiated in all slices throughout the cardiac cycle (turquoise)

CMR strain analysis was accomplished by an experienced, board-certified CMR cardiologist blinded to patient data and LGE images on a dedicated workstation using commercially available software (Caas MR Solutions, Version 5.2.2). After image registration, end-diastole and end-systole of the short and long-axis geometries were automatically determined by the software and corrected by the CMR cardiologist as needed. Automatic contouring of the endocardial and epicardial borders of the short-axis stack and the long axes (two- and four-chamber view) at end-diastole was manually corrected by the CMR cardiologist if required. Subsequently, automatic tracking of myocardial borders was initiated throughout the cardiac cycle and its acceptance by the CMR cardiologist allowed automatic calculation of MS. Global peak longitudinal strain (GLS), global peak circumferential strain (GCS) and global peak radial strain (GRS) as well as segmental peak longitudinal strain (SLS), segmental peak circumferential strain (SCS) and segmental peak radial strain (SRS) were automatically calculated by the software according to the AHA 16/17-segment model [28]. An exemplificative analysis is shown in Fig. 1.

### Statistical analysis

Categorical variables were summarized as counts (percentage), continuous variables as mean ± standard error. Linear and non-linear regression analysis was performed to evaluate the association of creatinine, urea and estimated glomerular filtration rate (eGFR) with MS values. In order to account for potential confounders such as LV-EF, scar burden, diabetes, hypertension, age, gender, LV mass or LV mass index, multiple linear regression models assessing the association between eGFR and GLS were developed.

Distribution of several parameters among predefined patient groups according to KDIGO stages [29] (for simplicity, patients without documented CKD were classified into the KDIGO 1 and 2 group according to their eGFR) was evaluated by analysis of variance (ANOVA). Post-hoc comparisons took place using LSD-test. Categorical variables were compared with Pearson-Chi-Quadrat test. The statistical test did not account for the interdependency of multiple segments among patients.

All statistical analyses were performed with SPSS software v 28.0 (IBM Corp., Armonk, NY, USA). Statistical significance was indicated by a *p* value < 0.05.

## Results

### Creatinine, urea and eGFR are associated with global myocardial strain in CMR

**Fig. 2:**
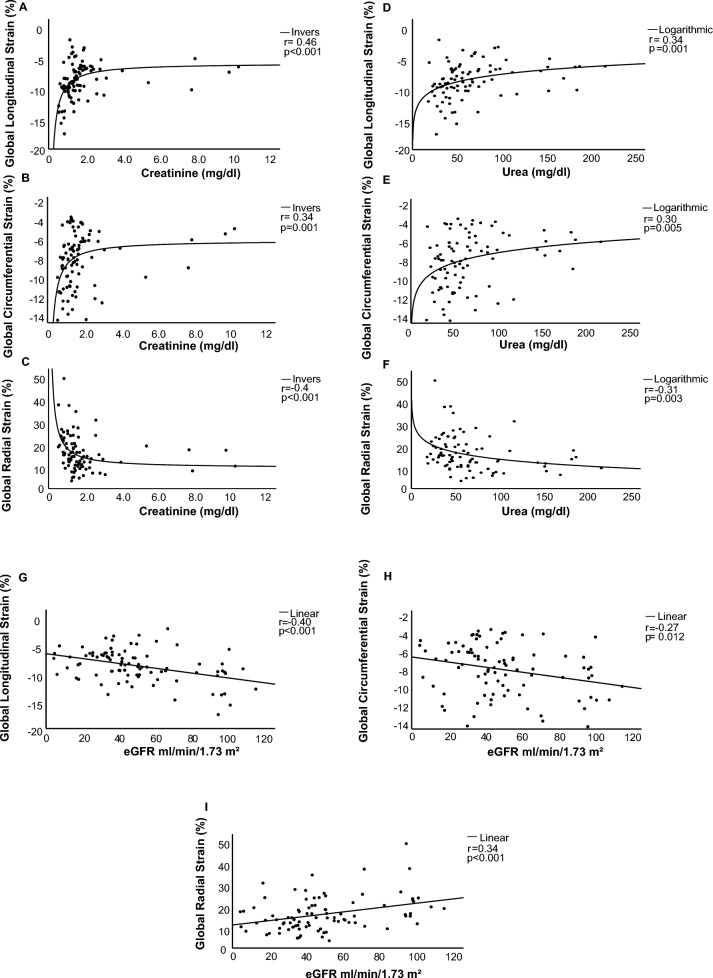
Creatinine, urea and eGFR are significantly associated with global myocardial strain in CMR. In patients with ICM, creatinine was significantly associated with GLS. These data were confirmed by both GCS as well as GRS (**A**–**C**). In addition to creatinine, urea (**D**–**F**) and eGFR (**G**–**I**) showed a significant association with GLS, GCS and GRS

Patient characteristics are reported in Table 1. In patients with ICM, creatinine was associated with myocardial function as determined by GLS (*r* = 0.46, *p* < 0.001). These data remained consistent also with regards to both GCS (*r* = 0.34, *p* = 0.001) and GRS (*r* = − 0.40, *p* < 0.001), as presented in Fig. 2A–C. Similarly, urea showed a significant association with GLS (*r* = 0.34, *p* = 0.001), GCS (*r* = 0.30, *p* = 0.005) and GRS (*r* = − 0.31, *p* = 0.003), as shown in Fig. 2D–F. Concordantly, eGFR was significantly associated with GLS (*r* = − 0.40, *p* < 0.001), GCS (*r* = − 0.27, *p* = 0.012) and GRS (*r* = 0.34, *p* < 0.001) (Fig. 2G–I).

**Table 1:** Patient characteristics

Patient characteristics	KDIGO 1, *n* = 14	KDIGO 2, *n* = 11	KDIGO 3, *n* = 47	KDIGO 4, *n* = 11	KDIGO 5, *n* = 6	*p* value
Age (years)	58.93 ± 1.55	67.09 ± 2.73	75.28 ± 1.06	76.18 ± 2.03	65.33 ± 7.23	< 0.001
Male sex (*n*, %)	11 (78.6)	9 (81.8)	37 (78.7)	8 (72.7)	6 (100)	0.760
HR (/min)	76.64 ± 3.24	70.64 ± 3.80	74.98 ± 2.23	75.91 ± 4.46	73.33 ± 5.13	0.86
BP (systolic) (mmHg)	123.86 ± 5.82	126.18 ± 25.38	125.16 ± 2.89	120.45 ± 7.87	113.17 ± 10.42	0.74
BP(diastolic) (mmHg)	76.21 ± 2.82	58.18 ± 13.47	68.64 ± 1.87	66.45 ± 3.82	56.67 ± 4.99	0.13
CAD-1 (*n*, %)	2 (14.3)	0 (0)	7 (14.9)	1 (9.1)	2 (33.3)	0.41
CAD-2 (*n*, %)	3 (21.4)	1 (9.1)	5 (10.6)	1 (9.1)	1 (16.7)	0.83
CAD-3 (*n*, %)	9 (64.3)	10 (90.9)	35 (74.5)	9 (81.8)	3 (50)	0.34
MRI parameters						
LV-EF (%)	41.57 ± 2.36	40.09 ± 2.81	33.91 ± 1.37	35.82 ± 2.7	38.67 ± 4.74	0.056
Scar burden (*n*,%)	5.79 ± 0.61	4.55 ± 0.72	4.87 ± 0.45	5.36 ± 0.94	3.0 ± 1.03	0.354
LV mass (g)	128.86 ± 6.10	142.36 ± 17.03	138.19 ± 4.19	132.55 ± 7.88	161.40 ± 20.85	0.412
LV mass index (g/m^2^)	66.14 ± 2.88	68 ± 6.38	71.43 ± 1.84	67.55 ± 3.85	80 ± 9.98	0.354
Cardiovascular risk profile						
Hypertension (*n*, %)	5 (41.7)	8 (72.7)	40 (93)	11 (100)	5 (83.3)	< 0.001
Current smoking (*n*, %)	7 (58.3)	5 (45.5)	9 (22.5)	1 (12.5)	3 (60)	0.049
Family history of CAD (*n*, %)	2 (16.7)	3 (27.3)	4 (10.5)	1 (11.1)	2 (40)	0.395
Diabetes (*n*, %)	4 (28.6)	6 (54.5)	23 (48.9)	7 (63.6)	4 (66.7)	0.397
Hyperlipidemia (*n*, %)	9 (75)	8 (80)	32 (78)	10 (100)	2 (40)	0.129
BMI (kg/m^2^)	25.49 ± 1.16	27.86 ± 1.30	27.30 ± 0.68	27.97 ± 1.23	26.68 ± 2.25	0.634
Apoplex/TIA	0 (0)	1 (9.1)	12 (25.5)	2 (20)	1 (20)	0.255
Prior myocardial infarction	5 (35.7)	4 (36.4)	21 (44.7)	2 (20)	2 (33.3)	0.688
NYHA class	1.67 ± 0.48	1.6 ± 0.75	2.23 ± 0.30	2.9 ± 0.78	3.25 ± 0.48	0.336
CVD	0 (0)	0 (0)	8 (17)	3 (30)	4 (66.7)	0.001
PAD/bowel ischemia	0 (0)	2 (18.2)	17 (36.2)	4 (40)	2 (40)	0.076
Lab values						
Creatinine (mg/dL)	0.76 ± 0.03	1.06 ± 0.04	1.53 ± 0.04	2.56 ± 0.12	7.58 ± 1.01	< 0.001
eGFR (ml/min/1.73 m^2^)	99.31 ± 1.65	69.88 ± 2.38	42.92 ± 1.16	22.25 ± 1.28	7.9 ± 1.52	< 0.001
Urea (mg/dL)	32.69 ± 2.69	38.73 ± 3.76	61.66 ± 3.80	123.0 ± 15.79	132.0 ± 20.72	< 0.001
Uric acid (mg/dL)	4.64 ± 0.40	6.55 ± 0.52	7.86 ± 0.70	8.80 ± 0.2	9.80 ±	0.002
Calcium, total (mmol/l)	2.22 ± 0.032	2.23 ± 0.06	2.26 ± 0.02	2.13 ± 0.04	2.17 ± 0.11	0.238
Phosphate (mmol/l)	1.01 ± 0.10	1.30 ± 0.20	1.24 ± 0.07	1.56 ± 0.12	1.79 ± 0.68	0.038
HbA1c (%)	5.76 ± 0.23	7.09 ± 0.57	6.80 ± 0.25	6.98 ± 0.47	7.1 ± 0.82	0.441
Cholesterol (mg/dL)	181 ± 19.86	138.33 ± 15.02	141.71 ± 6.67	145.63 ± 15.84	106.0 ± 10.43	0.054
LDLc (mg/dL)	139.71 ± 24.68	88.11 ± 13.93	84.72 ± 5.35	86.75 ± 10.85	51.67 ± 9.94	0.007
HDLc (mg/dL)	41.29 ± 1.82	39.78 ± 4.1	43.71 ± 2.33	43.88 ± 4.16	42.67 ± 10.81	0.940
Triglycerides (mg(dL)	80.13 ± 11.41	114.43 ± 19.39	121.05 ± 11.19	118.33 ± 15.65	78.67 ± 15.07	0.203
Medication						
Antiplatelet therapy (*n*, %)	12 (85.7)	9 (81.8)	37 (78.7)	10 (90.9)	5 (83.3)	0.905
Anticoagulation (*n*, %)	3 (21.4)	4 (36.4)	24 (51.1)	6 (54.5)	1 (16.7)	0.175
Statin (*n*, %)	14 (100)	10 (90.9)	44 (93.6)	11 (100)	6 (100)	0.678
Beta-Blocker (*n*, %)	10 (71.4)	10 (90.9)	43 (91.5)	10 (90.9)	5 (83.3)	0.371
ACE-inhibitors/ARBs (*n*, %)	10 (71.4)	8 (72.7)	35 (74.5)	9 (81.8)	5 (83.3)	0.963
Aldosterone antagonists (*n,* %)	3 (21.4)	4 (36.4)	22 (46.8)	4 (36.4)	1(16.7)	0.372
Loop diuretics (*n*, %)	4 (28.6)	5 (45.5)	36 (76.6)	11 (100)	4 (66.7)	< 0.001
Thiazide diuretics (*n*, %)	2 (14.3)	1 (9.1)	10 (21.3)	1 (9.1)	1 (16.7)	0.807
Neprilysin inhibitors (*n*, %)	1 (7.1)	2 (18.2)	7 (14.9)	2 (18.2)	0 (0)	0.692
SGLT2-inhibitors (*n*, %)	0 (0)	2 (18.2)	7 (14.9)	0 (0)	0 (0)	0.484

*HR* heart rate, *BP* blood pressure, *CAD* coronary artery disease, *PAD* peripheral arterial disease, *CVD* cerebrovascular disease, *TIA* transient ischemic attack, *LV-EF* left ventricular ejection fraction, *BMI* body mass index, *ACE* angiotensin converting enzyme, *ARBs* Angiotensin II receptor blockers, *SGLT2* Sodium-glucose cotransporter-2

### eGFR is significantly associated with segmental strain in CMR

**Fig. 3:**
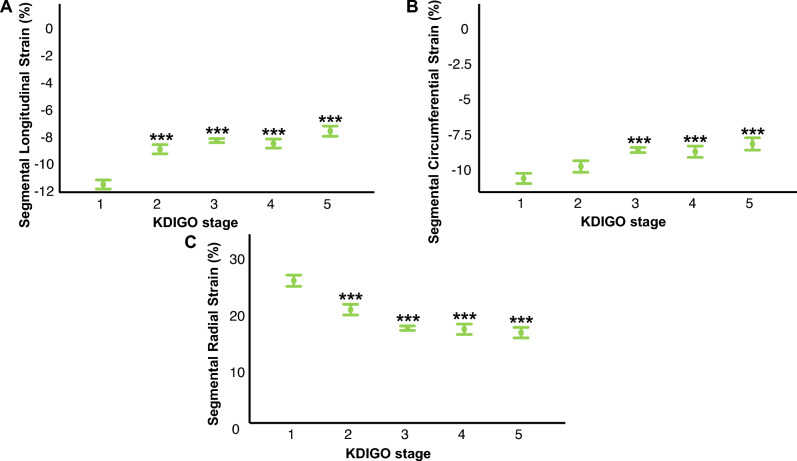
eGFR is significantly associated with segmental strain in CMR. A progressive reduction of SLS (**A**), SCS (**B**) and SRS (**C**) was observed with increasing severity of CKD, as expressed by KDIGO stages. *p ≤ 0.05, **p ≤ 0.01, ***p ≤ 0.001 for comparison with KDIGO-1

After demonstrating a significant association between renal function and global MS, we performed segmental MS analysis to further investigate the relationship between CKD und MS. SLS was progressively impaired with increasing severity of CKD, as expressed by the KDIGO-classification (KDIGO-1: − 11.93 ± 0.34; KDIGO-2: − 9.33 ± 0.34; KDIGO-3: − 8.69 ± 0.15; KDIGO-4: -8.91 ± 0.33; KDIGO-5: -7.99 ± 0.38; *p* < 0.001 for distribution, *p* < 0.001 for KDIGO-5 vs. KDIGO-1). Similar data were obtained for SCS and SRS. A graphic depiction of these data is shown in Fig. 3A–C.

**Fig. 4:**
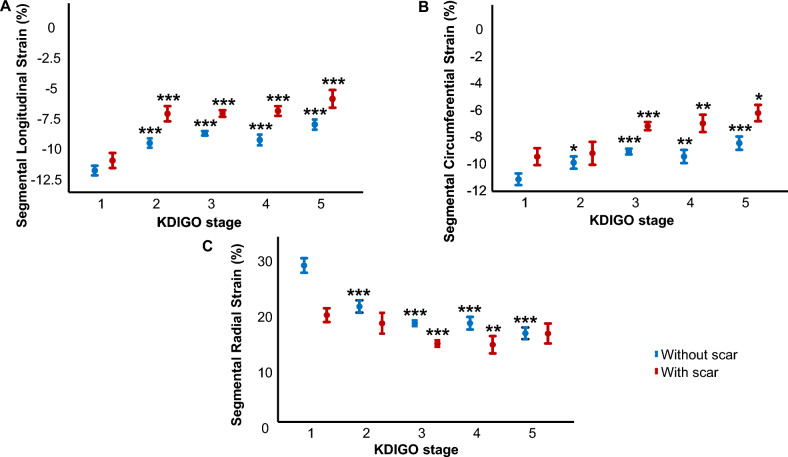
Impaired MS with increasing severity of CKD in patients with ICM is not due to presence or extent of myocardial scar. Segmental myocardial strain (SLS [**A**], SCS [**B**], SRS [**C**]) in patients with ICM was impaired with CKD in both scarred and non-scarred segments. *p ≤ 0.05, **p ≤ 0.01, ***p ≤ 0.001 for comparison with KDIGO-1 (respectively, scarred/non-scarred segments)

Furthermore, myocardial strain in CKD-patients with ICM was impaired in both scarred and non-scarred segments, as depicted in Fig. 4A–C.

### Increasing severity of CKD is independently associated with myocardial strain

As CKD was associated with impairment of MS, we investigated if this was an independent association and adjusted in a multivariable analysis for parameters with a known relationship with both CKD and MS.

Interestingly, eGFR was still significantly associated with GLS when adjusted for LV-EF (*p* < 0.001, Table 2, Model 1) and scar burden (*p* = 0.002, Table 2, Model 2). In addition, following further adjustment for clinical parameters with a known association with MS such as diabetes and hypertension, a decrease in eGFR was still significantly associated with an impaired GLS (*p* = 0.007, Table 2, Model 3). Besides, the association between eGFR and GLS was consistent even after further adjustment for age and gender (*p* = 0.003, Table 2, Model 4), LV mass (*p* = 0.008, Table 2, Model 5) or LV mass index (*p* = 0.007, Table 2, Model 6). These data demonstrate that the relationship between CKD and MS is not mediated by LV-EF, myocardial scar burden, diabetes, hypertension, age, gender, LV mass or LV mass index.

**Table 2:** Crude and adjusted models depicting linear association of renal function with GLS

Variable	*B*	*p*
Crude model		*R* = 0.340
eGFR	− 0.403	< 0.001

Variable	Adjusted *B*	*p*
Adjusted model N.1 (eGFR, LV-EF)		*R* = 0.782
eGFR	− 0.253	< 0.001
LV-EF	− 0.687	< 0.001
Adjusted model N.2 (eGFR, LV-EF, Scar burden)		*R* = 0.784
eGFR	− 0.230	0.002
LV-EF	− 0.719	< 0.001
Scar burden	− 0.071	0.361
Adjusted model N.3 (eGFR, LV-EF, Scar burden, Diabetes, Hypertension)		*R* = 0.798
eGFR	− 0.220	0.007
LV-EF	− 0.703	< 0.001
Scar burden	− 0.051	0.550
Diabetes	0.054	0.490
Hypertension	0.051	0.514
Adjusted model N.4 (eGFR, LV-EF, Scar burden, Diabetes, Hypertension, Age, Gender)		*R* = 0.808
eGFR	− 0.028	0.003
LV-EF	− 0.201	< 0.001
Scar burden	− 0.024	0.779
Diabetes	0.377	0.385
Hypertension	0.676	0.270
Age	− 0.029	0.210
Gender	0.677	0.220
Adjusted model N.5 (eGFR, LV-EF, Scar burden, Diabetes, Hypertension, Age, Gender, LV Mass)		*R* = 0.812
eGFR	− 0.026	0.008
LV-EF	− 0.191	< 0.001
Scar burden	− 0.003	0.976
Diabetes	0.472	0.288
Hypertension	0.494	0.435
Age	− 0.015	0.555
Gender	0.480	0.402
LV Mass	0.009	0.285
Adjusted model N.6 (eGFR, LV-EF, Scar burden, Diabetes, Hypertension, Age, Gender, LV Mass Index)		*R* = 0.809
eGFR	− 0.026	0.007
LV-EF	− 0.200	< 0.001
Scar burden	− 0.013	0.884
Diabetes	0.455	0.310
Hypertension	0.627	0.316
Age	− 0.026	0.269
Gender	0.608	0.280
LV Mass	0.007	0.687

*eGFR* estimated glomerular filtration rate, *LV* left ventricular, *LV-EF* left ventricular ejection fraction

## Discussion

The main finding of our study is that MS is significantly impaired with increasing severity of CKD in patients with ICM and in our cohort this finding was independent of LV-EF, the extent of myocardial scarring, diabetes, hypertension, age, gender, LV mass or LV mass index.

CKD is considered a serious global public health problem with an estimated global prevalence of 13.4% [7, 30, 31]. Deteriorating renal function has been shown to be associated with an exponential increase in all-cause and cardiovascular mortality. Thus, CKD represents one of the most relevant risk factors for the occurrence of CVD [7, 32]. The development of CVD in CKD-patients is not only related to traditional cardiovascular risk factors, but also to CKD-induced vascular and myocardial remodeling, resulting in myocardial fibrosis, hypertrophy and valve disease [7, 14]. In this context, CMR feature tracking to assess MS is an accurate and reliable non-invasive tool to analyze functional consequences of these myocardial changes. Previous studies already showed that increasing severity of CKD is associated with worsening MS in patients without a history of cardiovascular disease [25]. Remarkably, even in patients without known cardiovascular disease, an increasing number of cardiovascular risk factors, including CKD, has been shown to result in a decrease of GLS [33]. Furthermore, CKD further aggravates the impairment of LV strain in T2DM patients [23]. However, in the mentioned studies, patients with known cardiovascular disease and/or ICM were excluded. Thus, the relationship between CKD and MS specifically in patients with coronary artery disease and ICM due to prior myocardial infarction and scarring is still unclear. In this study, we extend the current knowledge by showing that in patients with known ICM, increasing severity of CKD significantly impairs ventricular contractility, reflected by worsening MS. This is demonstrated by the association of established markers of renal dysfunction—such as creatinine, urea and eGFR—with impairment of MS. Besides, this association was independent of clinical parameters with a known association with MS, such as diabetes, hypertension, LV mass or LV mass index as demonstrated by a persistent relationship between increasing severity of CKD and impaired MS following adjustment for the mentioned parameters in multivariable analyses.

Demonstrating a reduced myocardial contractility in patients with CKD explains on one hand at least partially the high prevalence of CVD in this population [1–5, 34]; on the other hand, it highlights the importance of early diagnosis, prevention and effective therapy of CKD in order to potentially avoid its effects on cardiovascular morbidity and mortality.

Once demonstrated that patients with CKD and ICM have a reduction of myocardial contractility which is directly proportional to the severity of their CKD stage, it is interesting to speculate about the mechanisms leading to this phenomenon. A possible explanation could be a more pronounced scar burden in patients with more advanced CKD, which may be due to accelerated atherosclerosis; in fact, it is well known that scarred segments present worsened MS compared to normal, viable myocardium [18, 21, 22]. However, in our study, cohort patients with higher stages of CKD did not show a more extensive left ventricular scar burden. Furthermore, the impairment of MS throughout stages of CKD was sustained even following adjustment for LV-EF and myocardial scar burden, suggesting that the negative effect of increasing severity of CKD on myocardial contractility may be at least in part independent of LV function or the extent of myocardial scarring. It is well known that MS is affected by scar transmurality, since transmurally scarred segments exhibit a more pronounced impairment of MS than non-transmural segments [35]. However, in the light of our findings, it is tempting to speculate that a more extensive myocardial scarring as a consequence of accelerated atherosclerosis may not be the key mechanism leading to the reduced myocardial contractility in patients with CKD.

A possible explanation may lay in a direct, non-atherosclerotic mediated effect of CKD itself on myocardial function. For instance, it is well known that CKD induces a systemic, chronic proinflammatory state [7]. The CKD-related release and accumulation of hormones, enzymes, cytokines and mediators as well as CKD-associated hemodynamic alterations are implicated in characteristic changes of the myocardium and the vasculature contributing to the high cardiovascular risk of CKD-patients [12, 16–23]. Besides, it is well known that CKD is associated with characteristic alterations of myocardial structure such as pathological myocardial fibrosis, with collagen deposition between capillaries and cardiomyocytes, and cardiac hypertrophy, leading to the development of uremic cardiomyopathy [7, 14]. Therefore, it is tempting to speculate that the ventricular dysfunction detected by impaired MS in our patient population reflects these CKD-related damages on the myocardium. However, typical parameters of myocardial fibrosis in CMR, such as T1 values and extracellular volume, were not available in our patient cohort, so that future studies are needed to further assess the role of myocardial fibrosis in the association between CKD and MS in patients with ICM.

Thus, CMR in general and more specifically MS may represent a useful tool in the assessment of CKD-related myocardial damage, possibly paving the way for the initiation and assessment of therapeutic strategies in the treatment of cardiovascular disease in patients with ICM and CKD, e.g. with high dose statins or potentially heart failure medications.

### Limitations

Although being, to the best of our knowledge, the first study analyzing the association between different stages of CKD and MS as determined by CMR in patients with ICM, our study population is relatively small and the statistical differences between KDIGO stages may be limited by low patient number. For example, the strain in KDIGO stage 5 was numerically impaired compared to KDIGO stage 3, but this did not reach statistical significance. Thus, our results need to be confirmed in larger study cohorts.

For patients on dialysis, eGFR may be imprecise due to the method of eGFR calculation**.**

Furthermore, our study focused on patients with ICM; consequently, further studies are required to analyze the effects of increasing severity of CKD on MS in patients with different types of cardiomyopathies. Due to the study design, we are unable to draw any conclusions regarding the prognostic impact of our results on patients with ICM and increasing severity of CKD. Finally, as previously described, absolute strain values are not generalizable, due to variability among vendors [35, 36].

## Conclusion

Myocardial strain is significantly impaired with increasing severity of CKD in patients with ICM. In our cohort, this relationship is independent of LV-EF, the extent of myocardial scarring, hypertension, diabetes, age, gender, LV mass or LV mass index and may be due to effects of CKD on myocardial function. Our study highlights the potential role of strain imaging as a screening tool in detecting CKD-related myocardial damage.

## Data Availability

The original contributions presented in this study are included in the article. Further inquiries can be directed to the corresponding author.

## References

[1] Ardhanari S, Alpert MA, Aggarwal K (2014) Cardiovascular disease in chronic kidney disease: risk factors, pathogenesis, and prevention. Adv Perit Dial 30:40–53 25338421

[2] Matsushita K, Ballew SH, Coresh J (2015) Influence of chronic kidney disease on cardiac structure and function. Curr Hypertens Rep 17(9):581 26194332 10.1007/s11906-015-0581-x

[3] Fujii H, Kono K, Nishi S (2019) Characteristics of coronary artery disease in chronic kidney disease. Clin Exp Nephrol 23(6):725–732 30830548 10.1007/s10157-019-01718-5 PMC6511359

[4] Bae EH et al (2012) GFR and cardiovascular outcomes after acute myocardial infarction: results from the Korea Acute Myocardial Infarction Registry. Am J Kidney Dis 59(6):795–802 22445708 10.1053/j.ajkd.2012.01.016

[5] Nakano T et al (2010) Association of kidney function with coronary atherosclerosis and calcification in autopsy samples from Japanese elders: the Hisayama study. Am J Kidney Dis 55(1):21–30 19765871 10.1053/j.ajkd.2009.06.034

[6] Major RW et al (2018) Cardiovascular disease risk factors in chronic kidney disease: a systematic review and meta-analysis. PLoS ONE 13(3):e0192895 29561894 10.1371/journal.pone.0192895 PMC5862400

[7] Jankowski J et al (2021) Cardiovascular disease in chronic kidney disease: pathophysiological insights and therapeutic options. Circulation 143(11):1157–1172 33720773 10.1161/CIRCULATIONAHA.120.050686 PMC7969169

[8] Wilson PW et al (1998) Prediction of coronary heart disease using risk factor categories. Circulation 97(18):1837–1847 9603539 10.1161/01.cir.97.18.1837

[9] Prevention C.f.D.C.a. (2017) National Chronic Kidney disease Fact Sheet. Department of Health and Human Services, Atlanta, GA

[10] Guerin AP et al (2000) Arterial stiffening and vascular calcifications in end-stage renal disease. Nephrol Dial Transplant 15(7):1014–1021 10862640 10.1093/ndt/15.7.1014

[11] London GM, Guerin AP (1999) Influence of arterial pulse and reflected waves on blood pressure and cardiac function. Am Heart J 138(3 Pt 2):220–224 10467216 10.1016/s0002-8703(99)70313-3

[12] Gauthier-Bastien A et al (2014) Vascular remodeling and media calcification increases arterial stiffness in chronic kidney disease. Clin Exp Hypertens 36(3):173–180 23786435 10.3109/10641963.2013.804541

[13] Salib M et al (2022) Serum markers of fibrosis, cardiovascular and all-cause mortality in hemodialysis patients: the AURORA trial. Clin Res Cardiol 111(6):614–626 34170371 10.1007/s00392-021-01898-9 PMC9151553

[14] Alhaj E et al (2013) Uremic cardiomyopathy: an underdiagnosed disease. Congest Heart Fail 19(4):E40–E45 23615021 10.1111/chf.12030

[15] Shah RV, Abbasi SA, Kwong RY (2014) Role of cardiac MRI in diabetes. Curr Cardiol Rep 16(2):449 24430012 10.1007/s11886-013-0449-0 PMC3965673

[16] Negishi K et al (2013) Independent and incremental value of deformation indices for prediction of trastuzumab-induced cardiotoxicity. J Am Soc Echocardiogr 26(5):493–498 23562088 10.1016/j.echo.2013.02.008

[17] Negishi K et al (2014) Use of speckle strain to assess left ventricular responses to cardiotoxic chemotherapy and cardioprotection. Eur Heart J Cardiovasc Imaging 15(3):324–331 24057661 10.1093/ehjci/jet159

[18] Polacin M et al (2022) Segmental strain for scar detection in acute myocardial infarcts and in follow-up exams using non-contrast CMR cine sequences. BMC Cardiovasc Disord 22(1):226 35585495 10.1186/s12872-022-02664-z PMC9118592

[19] Claus P et al (2015) Tissue tracking technology for assessing cardiac mechanics: principles, normal values, and clinical applications. JACC Cardiovasc Imaging 8(12):1444–1460 26699113 10.1016/j.jcmg.2015.11.001

[20] Pedrizzetti G et al (2016) Principles of cardiovascular magnetic resonance feature tracking and echocardiographic speckle tracking for informed clinical use. J Cardiovasc Magn Reson 18(1):51 27561421 10.1186/s12968-016-0269-7 PMC5000424

[21] Richardson WJ et al (2015) Physiological implications of myocardial scar structure. Compr Physiol 5(4):1877–1909 26426470 10.1002/cphy.c140067 PMC4727398

[22] Stathogiannis K et al (2020) Regional myocardial strain by cardiac magnetic resonance feature tracking for detection of scar in ischemic heart disease. Magn Reson Imaging 68:190–196 32084516 10.1016/j.mri.2020.02.009

[23] Zhang Y et al (2021) The additive effects of kidney dysfunction on left ventricular function and strain in type 2 diabetes mellitus patients verified by cardiac magnetic resonance imaging. Cardiovasc Diabetol 20(1):11 33413395 10.1186/s12933-020-01203-4 PMC7792094

[24] Terhuerne J et al (2021) Speckle-tracking echocardiography in comparison with ejection fraction for prediction of cardiovascular mortality in patients with end-stage renal disease. Clin Kidney J 14(6):1579–1585 34276976 10.1093/ckj/sfaa161 PMC8280917

[25] Jia X et al (2022) Cardiac magnetic resonance imaging parameters show association between myocardial abnormalities and severity of chronic kidney disease. Front Cardiovasc Med 9:1053122 36465471 10.3389/fcvm.2022.1053122 PMC9712745

[26] Weinreb JC et al (2021) Use of intravenous gadolinium-based contrast media in patients with kidney disease: consensus statements from the American College of Radiology and the National Kidney Foundation. Kidney Med 3(1):142–150 33604544 10.1016/j.xkme.2020.10.001 PMC7873723

[27] Schulz-Menger J et al (2020) Standardized image interpretation and post-processing in cardiovascular magnetic resonance—2020 update : Society for Cardiovascular Magnetic Resonance (SCMR): Board of Trustees Task Force on Standardized Post-Processing. J Cardiovasc Magn Reson 22(1):19 32160925 10.1186/s12968-020-00610-6 PMC7066763

[28] Cerqueira MD et al (2002) Standardized myocardial segmentation and nomenclature for tomographic imaging of the heart. A statement for healthcare professionals from the Cardiac Imaging Committee of the Council on Clinical Cardiology of the American Heart Association. Int J Cardiovasc Imaging 18(1):539–542 12135124

[29] Levey AS et al (2020) Nomenclature for kidney function and disease-executive summary and glossary from a Kidney Disease: Improving Global Outcomes (KDIGO) consensus conference. Eur Heart J 41(48):4592–4598 33141221 10.1093/eurheartj/ehaa650 PMC7774468

[30] Eckardt KU et al (2013) Evolving importance of kidney disease: from subspecialty to global health burden. Lancet 382(9887):158–169 23727165 10.1016/S0140-6736(13)60439-0

[31] Hill NR et al (2016) Global prevalence of chronic kidney disease—a systematic review and meta-analysis. PLoS ONE 11(7):e0158765 27383068 10.1371/journal.pone.0158765 PMC4934905

[32] Tonelli M et al (2006) Chronic kidney disease and mortality risk: a systematic review. J Am Soc Nephrol 17(7):2034–2047 16738019 10.1681/ASN.2005101085

[33] Takahashi T et al (2022) Association between cardiovascular risk factors and left ventricular strain distribution in patients without previous cardiovascular disease. J Echocardiogr 20(4):208–215 35562627 10.1007/s12574-022-00576-7 PMC9106265

[34] Dilsizian V et al (2021) Cardiac imaging for coronary heart disease risk stratification in chronic kidney disease. JACC Cardiovasc Imaging 14(3):669–682 32828780 10.1016/j.jcmg.2020.05.035

[35] Cimino S et al (2013) Global and regional longitudinal strain assessed by two-dimensional speckle tracking echocardiography identifies early myocardial dysfunction and transmural extent of myocardial scar in patients with acute ST elevation myocardial infarction and relatively preserved LV function. Eur Heart J Cardiovasc Imaging 14(8):805–811 23258316 10.1093/ehjci/jes295

